# Research Progress on Plant Responses to Stress Combinations in the Context of Climate Change

**DOI:** 10.3390/plants13040469

**Published:** 2024-02-06

**Authors:** Zeyao Jing, Na Liu, Zongxian Zhang, Xiangyang Hou

**Affiliations:** 1 College of Grassland Science, Shanxi Agricultural University, Jinzhong 030801, China; jing_zeyao@163.com (Z.J.); liuna720@yeah.net (N.L.); zongxian.zhang1@gmail.com (Z.Z.); 2 Key Laboratory of Model Innovation in Forage Production Efficiency, Ministry of Agriculture and Rural Affairs, Jinzhong 030801, China

**Keywords:** plants, stress combinations, multifactorial stress combinations, research progress, methods

## Abstract

In the context of climate change, the frequency and intensity of extreme weather events are increasing, environmental pollution and global warming are exacerbated by anthropogenic activities, and plants will experience a more complex and variable environment of stress combinations. Research on plant responses to stress combinations is crucial for the development and utilization of climate-adaptive plants. Recently, the concept of stress combinations has been expanded from simple to multifactorial stress combinations (MFSCs). Researchers have realized the complexity and necessity of stress combination research and have extensively employed composite gradient methods, multi-omics techniques, and interdisciplinary approaches to integrate laboratory and field experiments. Researchers have studied the response mechanisms of plant reactive oxygen species (ROS), phytohormones, transcription factors (TFs), and other response mechanisms under stress combinations and reached some generalized conclusions. In this article, we focus on the research progress and methodological dynamics of plant responses to stress combinations and propose key scientific questions that are crucial to address, in the context of plant responses to stress assemblages, conserving biodiversity, and ensuring food security. We can enhance the search for universal pathways, identify targets for stress combinations, explore adaptive genetic responses, and leverage high-technology research. This is in pursuit of cultivating plants with greater tolerance to stress combinations and enabling their adaptation to and mitigation of the impacts of climate change.

## 1. Introduction

Over the past few decades, numerous anthropogenic activities have led to an increase in greenhouse gas emissions, leading to an increase in the global temperature. Within the context of climate change, the frequency, intensity, breadth, and simultaneity of extreme weather events—such as heat waves, excessive rainfall, droughts, and tropical cyclones—have increased [1]. Extreme weather events are characterized by abruptness, unpredictability, and devastating impact [2] and adversely affect global food security, water supply and demand, ecosystem productivity, and the global carbon cycle. Research indicates that each region will face concurrent and compounded impacts from climate drivers more frequently, with a heightened occurrence of combined extreme weather events, such as heat waves and droughts [3]. The repercussions of compounded weather events will be more hazardous and unpredictable.

Extreme weather events have significantly increased the number and intensity of stressors on plants, combined with poor soil conditions, limited nutrient availability, different human-made pollutants and radiation, and attacks by different pathogens or insects [4]. The unpredictable intertwining of these biotic and abiotic stressors, affecting plants and microbiomes concurrently or successively, elevates the intricacy of the forthcoming plant environment. Consequently, this complexity results in drastic declines in plant growth, yield, overall health, and the biodiversity of the pivotal microbiomes on which plants rely [5,6].

Under natural conditions, plants are frequently subjected to a combination of two or more environmental stresses, which increases their complexity and uncertainty. The amalgamation of these stressors often poses more complex threats to agricultural production than singular stresses and is challenging to predict [7,8]. There are many stress factors in nature that affect plant growth, and there are synergistic, antagonistic, or cumulative interactions among these factors. Moreover, the consequences of stress combinations hinge upon the plant’s developmental stage and the type, intensity, and timing of the stresses [9,10]. Furthermore, analyses of stress combinations and their individual stresses at the transcriptional and metabolic levels reveal that stress combinations trigger specific pathways in plants that differ from those activated by singular stresses. Additionally, cross-communication occurs among signal transduction pathways and the interplay of various metabolic components and complexities cannot be directly inferred from a plant’s response to individual stresses [11,12,13]. Recent studies have revealed that as the quantity and intricacy of stresses affecting plants concurrently rise, the survival of plants and ecosystem processes will drastically decline, even with a relatively low level of each stress [14]. This indicates that occasional increases in low-level stress sources in a low-level stress combination environment may have rapid and severe effects on plant growth and ecosystems. Therefore, research on plant responses to stress combinations in the context of climate change is particularly important.

In the past, most research focused on plant responses to a single stress, whereas research on plant responses to combinations of two or more stresses has increased in recent decades, based on current natural production needs and future climate change considerations [15]. The concept of stress has changed from the original concept to that of stress combinations. The concept of stress combinations has also expanded from simple combinations of two or at most three stressors to the current multifactorial stress combinations (MFSCs) involving three and more stressors that affect plants simultaneously or sequentially [6]. The research focus on plants under stress combinations has evolved, delving beyond phenotypic observation, growth and physiological indices, and related gene detection. It now encompasses microscopic alterations in plant morphology, studies on physiological pathways, modes of action of functional genes, and the cultivation of specific varieties. This study aimed to consolidate current research focuses and methodologies regarding plant responses to stress combinations. Furthermore, it outlines future research trends aimed at enhancing our understanding of stress combinations.

## 2. Definition of Stress Combination

Stress combinations describe the simultaneous exposure of plants to two or more stresses [11,12]. In the past, stress combinations were defined as simple combinations of two or at most three different stresses, and extensive research has been conducted in this realm [16,17,18]. Given the escalating environmental complexity arising from heightened levels of various pollutants and erratic weather patterns due to climate change, studies indicate a substantial decline in plant growth and survival even when each stressor is at a relatively low level, as the number of stressors amplifies their effects [14]. Therefore, studying plant responses to basic stress combinations alone is inadequate for intricate environments.

Therefore, the concept of stress combinations has recently broadened, introducing a novel approach to studying plant responses to combined stresses termed “multifactorial stress combinations”. This term denotes combinations of three or more (*n* ≥ 3) stressors affecting the plant simultaneously or in succession. The simultaneous impacts on plants come from a multitude of stressors such as anthropogenic and non-anthropogenic biotic factors, climatic drivers, and soil-related abiotic factors [6,19,20].

Stress combinations include both simple stress combinations and MFSCs, which affect plant growth, development, and reproduction. Simple stress combinations were studied in the context of the present environmental conditions, whereas MFSCs were investigated in complex and uncertain future environmental contexts. Both these avenues warrant sustained and thorough research.

## 3. Current Research Focus

### 3.1. Simple Stress Combinations

#### 3.1.1. Abiotic Stress Combinations

Several scholars have focused on stress combinations and conducted a great deal of research. Many scholars have focused on abiotic stress combinations to address specific stress combinations relevant to their crop or region of interest. These include staple crops such as *Zea mays* and *Glycine max* under a combination of drought and heat stress [11], and trees in the Mediterranean region, which are vulnerable to drought and acute ozone [21]. Numerous studies have highlighted the profound effects of stress interactions on plants. Combinations of drought and heat waves, drought and ozone, or high temperature and salt have been found to have significantly more pronounced negative effects on plants than individual stress components applied in isolation [21,22,23,24]. Studies have also highlighted the positive effects of the interplay between two distinct stresses on plants. For instance, mild salt addition alleviates plant damage caused by low-temperature stresses [10,25].

The coupling of drought and high-temperature stress has led to a catastrophic decline in agricultural productivity. Consequently, more research has focused on understanding the effects of combined high temperatures and drought on plant growth and productivity [26,27]. Recent studies have revealed that the convergence of drought and high-temperature stress has a more detrimental effect on plant and crop growth than either stress alone. This synergy significantly diminished crop yield, indicating a shared defense mechanism across various plants to compete with the combined challenges of drought and high temperatures [8]. Nevertheless, various plants exhibit distinct responses to stress combinations in terms of the degree and manner of impact. For instance, *Arabidopsis thaliana* demonstrated greater susceptibility to the combined stress of salt and high temperatures than *Solanum lycopersicum* [28]. Cereals try to compensate for yield losses during combined drought and high-temperature stress by greater reductions of nutrient growth and seed size compared to legumes [29,30]. The impact on numerous vital food crops intensifies notably when combined drought and high-temperature stresses occur during a plant’s reproductive stages [24,26,30]. Recent studies have highlighted that the sequence in which drought and high-temperature stresses are applied does not change the plant’s response to combined stress. The metabolism of reactive oxygen species (ROS) and stomatal responses are pivotal in the adaptation of plants to combinations of drought and high-temperature stress [10].

#### 3.1.2. Abiotic and Biotic Stress Combinations

Amid climate change and heightened environmental intricacies, the intersection of abiotic and biotic stresses has become a key research focus. Numerous studies investigating combined stresses delve into the interplay between abiotic and biotic stressors, like the interactions between drought and pests, cadmium and pathogens, and drought and pathogens [31,32,33,34]. Reports are available indicating that certain abiotic stress conditions, such as ozone stress, can bolster plant resilience against pathogen attacks in certain cases. However, in most scenarios, prolonged exposure of plants to abiotic stresses, such as drought or nutrient deprivation, tends to weaken their defenses, making them more susceptible to pests or pathogens, such as arthropods [11,33,35]. Abiotic stresses play a pivotal role in modulating plant tolerance or susceptibility to pathogens through various mechanisms, resulting in altered plant–pathogen interactions, influenced by factors such as plant species, pathogen type, and stress intensity [23].

### 3.2. Multifactorial Stress Combinations

Several abiotic stresses occur simultaneously, which is more lethal to crops than specific stress conditions [11]. The escalating impact of climate change and environmental pollution has correspondingly heightened the multitude and intricacy of stresses encountered by plants [36]. The recent emergence of MFSCs has focused on the response of plants exposed to three or more stress combinations simultaneously or sequentially [20], which has yielded some important conclusions.

Studies investigating MFSCs in *Arabidopsis thaliana*, *Oryza sativa*, and *Zea mays* have revealed substantial detrimental effects on plants. These findings indicate that even when individual stress levels are relatively low, the cumulative effect significantly diminishes plant growth and survival as the number of stressors increases [4,19]. Additional studies have demonstrated that plants navigate multifaceted stress combinations through distinct pathways and specialized processes [4]. Analysis of various *Oryza sativa* genotypes reveled noteworthy genetic diversity within its response mechanism [19]. As the number of stressors in a multifactorial stress combination increased, a consistent trend was observed in plant soil and microbial communities. This trend is aligned with the increasing number of stress factors, showing a notable decline in diversity [5,20].

### 3.3. Response Mechanisms to Stress Combinations

#### 3.3.1. Reactive Oxygen Species

ROS homeostasis plays a crucial role in plant survival under stress combinations, and mutants with impaired ROS regulation exhibit high sensitivity to stress combinations [4,13]. In response to stress combinations, ROS function as pivotal signaling molecules, enabling the rapid detection of various stimuli and activation of regulatory pathways, including stomatal movement, abscisic acid (ABA), and immune responses [17]. This adjustment aids in adapting coping strategies, establishing defense mechanisms, and restoring growth capacity [37,38]. Despite their role as signaling molecules, ROS are also toxic by-products of stress metabolism. Elevated ROS levels can initiate genetically programmed cell death [12,39]. Plants exhibiting higher antioxidant capacities or lower ROS accumulation typically demonstrate greater resilience to stress combinations [10]. Effective detoxification of ROS is assumed to play a key role in enhancing plant tolerance to stress combinations [36]. Recent studies on plant ROS during stress combinations have revealed that while ROS can benefit plants amidst abiotic stresses, this is contingent on cells maintaining sufficiently high energy reserves to detoxify ROS, which enables plants to modulate their metabolism and craft suitable adaptive responses [12]. Following exposure to stress combinations, components such as flavanols [36], amino acids, and polyamines [40] show heightened accumulation in plants. They act as antioxidants, preserve cellular ROS homeostasis, and mitigate plant damage caused by stress combinations.

ROS waves are another aspect of plant response to stress combinations. ROS are produced as signaling molecules after stress in plants and are coupled with Ca^2+^ and electrical signals to form rapid and widespread systemic signals called ROS waves [41,42]. The ROS wave transmits signals to neighboring or distant cells, collaborating with various signaling components to orchestrate a systemic response. This process effectively regulates how plants respond to stress combinations [43], during which plants can integrate various systemic signals, generated simultaneously, through ROS waves. These signals can be swiftly transmitted from damaged parts, whether the same or different, to the entire plant within a few minutes [44]. The speed and efficiency of ROS signaling are correlated with the specific site of damage [45]. ROS waves are considered crucial signals that traverse through plant vascular bundles and chloroplasts [46]; ROS waves serve as a warning system for cells and tissues, signaling imminent stress. They are often accompanied by other signals that may convey specificity, eliciting systemic acquired acquisitiveness (SAA) to safeguard against growth and defense responses. Notably, plants without ROS waves do not exhibit SAA [43]. Thus, ROS waves are crucial for plant responses to stress combinations.

#### 3.3.2. Plant Hormones

Plant hormones are other signaling molecules that plants use in response to stress combinations, coordinating multiple signal transduction pathways under stress combinations [47]. They play key roles in plant responses to stress combinations [48]. Various combinations of stress trigger distinct physiological and molecular responses in plants. These responses lead to alterations in the phytohormone and ROS levels, which subsequently influence each other [12]. Therefore, the ability of phytohormones to regulate antioxidant defense systems may be crucial for plant adaptation to stress combinations [27,47]. Hormones such as ABA, jasmonic acid (JA), salicylic acid (SA), and melatonin (MET) have previously been identified to play important roles in plant responses to stress combinations [49].

ABA is a major hormone in plant responses to stress combinations, regulating stomata and altering adaptor protein expression [50], which plays an important role in plant adaptation to stress combinations [27]. ABA serves as a pivotal regulator, controlling various response networks and bolstering plant resilience against stress combinations [51]. For example, ABA plays a crucial role in regulating the accumulation of vital proteins during high temperature and drought stress combinations [52]. Mutants deficient in ABA metabolism and signaling exhibit higher susceptibility than wild-type plants to combinations of salinity and high temperature [28], as well as salinity and intense light stress combinations [49]. ABA accumulation is affected by stress combinations [31], and interactions with ROS have important effects on plant adaptation to stress combinations [12,28].

Various stresses within a combination trigger hormone-signaling interactions. Other hormones, such as JA, SA, and MET, also participate in plant responses to stress combinations, contributing significantly to systemic signaling integration in plants [45]. JA plays a key role in the plant response of *Arabidopsis thaliana* to the combination of high-light and high-temperature stresses [53], and SA mitigates the damaging effects of combined drought, high-temperature, and salinity stresses by improving the antioxidant system [54]. Conversely, MET may function primarily as an antioxidant under stress combinations [55,56]; increased levels of MET under salinity and heat stress combinations enhance ROS detoxification and improve the acclimatization of *Solanum lycopersicum* by specifically regulating the expression of antioxidant-related genes, and the exogenous application of MET achieves a similar effect [39].

#### 3.3.3. Transcription Factors

TFs are pivotal in the regulation of transcriptional processes, have broad involvement in plant growth and development, and are crucial in responding to stress combinations. Employing TFs to modulate the expression of specific genes proves to be an effective strategy for inducing plant tolerance [57,58,59]. Recent studies on *Arabidopsis thaliana* and *Zea mays*, under stress combinations, have revealed that these combinations induce distinct transcriptional changes that cannot be anticipated by plant responses to individual stressors [8,29]. In addition, transcriptome analyses of different soybean tissues under drought and high-temperature stress combinations showed that each tissue responded differently to the stress combinations [60].

Studies on *Oryza sativa*, *Helianthus annuus*, and *Triticum aestivum* have shown that plants express specific genes under stress combinations compared to single stresses, and that the responses to different stress combinations can be regulated by specific TFs [15,61,62]. Nevertheless, recent studies on several different stress combinations involving heat have shown that some TF families, such as heat shock factors (HSFs), myeloblastosis (MYB), and ethylene response factors (ERFs), may be used to enhance plant tolerance to different types of stress combinations involving heat when stress combinations with the same factor are involved [63,64,65]. These TFs play unique roles in stress combinations, whether they are specific TFs in stress combinations or common to different stress combinations, and in-depth studies on their own functions and regulatory pathways may be an effective means to reveal the regulatory pathways of stress combinations.

## 4. Research Method

### 4.1. Composite Gradient Method

The major methodology used to study the effects of stress combinations was the controlled variable approach, in which the stress combination was in a controlled state, whereas other factors remained unmanipulated [60,62]. With this design, the effect of each stress combination on the plant was estimated [66,67]. Many combinations of stresses that could have a significant impact on agricultural production have been summarized in a “stress matrix”; however, each combination is limited to two stresses [10,11].

This design increases in cost with the number of stressors; therefore, the number of studies on the effects of multiple stressors is sparse. Rillig et al. surveyed soil-based global change-driver experiments and noted that very few of these experiments (<2%) involved three or four drivers [5]. To investigate the effects of MFSCs, Rillig et al. drew inspiration from the effects of biodiversity on ecosystem functioning by composing a pool of 10 stressors, from which they randomly chose a gradient of an increasing number of factors and found a severe decline in plant survival and microbial community biodiversity, showing a consistent directional trend along the number of factors [5]. The design of this study is generally applicable to ecosystems facing MFSCs [7].

Recent studies proposed that stress combinations represent distinct states in plants under stress. Within these combinations, plants can modulate this state through the interplay of diverse signaling pathways, triggering specific molecular responses such as distinctive promoter models, transcription factor binding sites, and transcripts [15]. These interactions lead to the emergence of novel defenses or domestication responses that surpass the simple cumulative effects of multiple stresses [8,11]. This perspective could signify a novel approach for exploring plant responses to stress combinations.

### 4.2. Multi-Omics Approach

Recently, genomic technology has made great progress, and the use of genomics, transcriptomics, proteomics, metabolomics, and other histological methods has become an important means for studying the response mechanisms of plants to stress combinations [8,40,68,69]. Transcriptomic and genome-wide association studies (GWASs) have revealed specific transcript changes in plants experiencing stress combinations, a substantial portion of which cannot be extrapolated from individual stress conditions [13,28,53]. Most of these specific transcripts indicate that plant responses to stress combinations are unique [23,36]. In stress combinations, the majority of transcripts show additive effects between two different stresses, and a small proportion show antagonistic responses [63,70]. Analyses of the plant metabolome have shown that sucrose replaces proteins as the major osmotic protectant in plants under drought and heat stress combinations [29].

With the continuous advancement of histological techniques, integrated multi-omics analysis has become an indispensable component of systems biology [71]. Compared with single-omics approaches, integrating multi-omics data enables the comprehensive characterization of plants facing stress combinations across multiple scales. This integration enhances the efficiency of screening traits related to stress combination tolerance and facilitates a thorough analysis of the intricate regulatory network governing plant responses to these stress combinations [72,73]. Transcriptomic and proteomic analyses in several plant species indicate that antioxidant defense mechanisms play an important role under stress combinations [10,74]. Proteomic and metabolic analyses have shown that malate metabolism plays an important role in the response of *Arabidopsis thaliana* to stress combinations under drought and heat stress combinations [75]. Using historical technology and integrating and mining multi-omics data may provide more ideas for studying plant response mechanisms under stress combinations.

### 4.3. Integration of Laboratory and Field Ecosystems

Over the past few decades, notable progress has been made in studying the responses of plants to stress combinations under controlled laboratory conditions [28,76]. However, many laboratory experiments on plant responses to stress combinations are limited in accurately simulating field environments [77]. Achieving consistency between plant responses observed in laboratory settings and those in field ecosystems is crucial. Field environments are remarkably more complex and unpredictable than controlled laboratory conditions. In field settings, plants often encounter heterogeneous conditions [78], and they may face concurrent or sequential occurrences of stresses, contaminants, pests, and wildlife. Consequently, plants are more vulnerable to combinations of stressors [79].

Current field studies examining plants under different stress combinations have suggested that most of these combinations exhibit synergistic effects that significantly affect plant growth and development. These effects may be more pronounced in ecosystems with lower biodiversity, such as farmlands [20]. The interaction between genotype and environment makes the responses of different varieties to stress combinations variable, and high-yielding varieties that are more stable to stress combinations must be selected [80]. Consequently, although laboratory experiments should be carefully designed to better emulate field conditions [23], combining both laboratory and field experiments is imperative to address specific crops and stress combinations that pose a threat to food security.

### 4.4. Integrated Application of Multidisciplinary Technology

The current application of multidisciplinary techniques in integrated research also plays an increasingly important role in understanding the responses of plants to stress combinations [13]. Plant nanobiotechnology is an emerging field that aims to regulate plant stress responses [81], and several nanomaterials have shown promise in enhancing the resistance of crops, such as *Gossypium herbaceum* [81] and *Oryza sativa* [82], to challenging environmental conditions. Nanomaterials can enhance plant resistance by enhancing ROS-activated stress signaling pathways, regulating the expression of defense genes and stomatal status, increasing the efficiency of PSII response centers, and stress-training plants to enhance resistance [38,82,83]. Genome editing technologies such as CRISPR/Cas9 are also important tools for studying plant responses to stress combinations [84], which allow editing of the genome in a highly accurate way to obtain desired traits [85], and have been successfully applied to a wide range of plants such as cereals [86] and fruit trees [87], as well as to pest management [88].

Stress combinations are common problems faced by organisms. We can learn from the experience and knowledge of other disciplines, broaden our research ideas and methods, and alleviate the adverse effects of stress combinations on plants [14]. The concept of the MFSC was inspired by ecology and yielded important conclusions on plant responses to stress combinations, illustrating the use of plant–microbe interactions to mitigate the adverse effects of multiple stresses on plants [20,89]. Proteins that regulate iron and ROS levels in cells play important roles when prokaryotes and animals are exposed to extreme environments [90]. Recent studies have shown that the management of iron and ROS levels in MFSCs is critical for plant survival [4].

## 5. Future Research Directions

### 5.1. Key Scientific Issues

The following areas of research could be strengthened in terms of plant responses to multiple stressors. 1. Finding key nodes or thresholds: Previous studies have identified some of the important mechanisms of plant responses to stress combinations [17,24] and observed that with an increase in stress types in the stress combination, plant survival plummets [14], providing a preliminary understanding of the plant responses to stress combinations. However, few studies have been conducted on the thresholds for plant survival under stress combinations and the critical nodes that lead to plant death, and quantitative metrics are scarce for the extent of stress combinations. Further research on related aspects is of great importance to judge the degree of stress and predict the plant state. 2. Systematically integrated existing data to further study the key mechanisms of plant responses to stress combinations: Many studies have been conducted on the responses of plants to stress, and large amounts of data have been obtained. Stress combinations, as a special state of plants facing unfavorable environments, and the research results of plant responses to a single stress, have important reference significance. However, the mining and utilization of these data are not sufficiently deep, and research on the key mechanisms of plant responses to stress combinations needs to be strengthened. 3. Finding common and efficient research methods: The stress combinations experienced by plants are complex. Currently, few research methods are available on plant responses to stress combinations, and most follow previous research methods. However, owing to the increase in the number of variables involved, the research costs are high; therefore, progress is slow [5]. Efficient research methods can accelerate the study of plant responses to stress combinations, so that we can better cope with climate change.

Under the complex climate conditions of the future, plants are likely to experience multiple stressful environments more frequently, which presents novel challenges and directions for conserving biodiversity and ensuring food security. 1. Exploring plant perceptions and prioritization of responses to stress combinations: Studies have shown that one stressor will dominate under a stress combination, and plants will preferentially sense or respond to this primary stressor and react to it, involving complex signaling pathways [91]. The study of relevant pathways in plants will help us understand plant response mechanisms. 2. Selection and breeding of multi-resistant varieties of plants: With climate change, the frequency and intensity of plant stress combinations are increasing, and it is particularly important to develop varieties that are more resilient to multiple stresses [92]. Wild plant germplasm resources can be collected and used, and the molecular and physiological mechanisms of plants that can grow well under multiple stresses or those that are not sensitive to stress can be explored, to improve the resistance of plants to multiple stresses. 3. Novel agroecosystems: In the future, complex climate changes may pose a threat to food security, and novel and more suitable agroecosystems and models are needed [93]. This requires enhanced research in a number of areas under stress combination conditions, including plant–microbe interaction research, plant mixing, crop rotation methods, and the development of novel plant fertilizers.

### 5.2. Research Entry Point

#### 5.2.1. Finding Common Pathways

Recently, by comparing the responses of plants under combined and single stresses, a dominant factor was observed in the responses of plants to stress combinations, with the response to one stress being prioritized over the other [40]; physiological and molecular processes are also similarly responsive to the dominant stress source [23]. Using the transcriptome, metabolome, and proteome, it was found that under stress combinations, plants produce common responses independent of the type of stress and specific responses related to the type of stress combination, with a significant overlap of TFs and signaling pathways in the signal transduction pathways [8,9,29,62,94]; however, there are differences in gene expression levels [23]. In addition, commonalities among different stress combinations have been found by comparing their transcriptomes [27]. This suggests that common pathways may exist in plants in response to stress combinations; however, the expression level is related to the stress combination itself.

Simultaneously, stress combinations evoke a considerable number of specific responses. As the number of stresses within a combination increases, the complexity of plant reaction intensifies. Some typical responses observed under individual stress conditions may be suppressed, whereas the number of gene expression responses unique to the stress combination may increase [4].

Therefore, it is plausible to pinpoint the dominant factors and central hubs of plant responses to stress combinations by scrutinizing shared responses (between two single stresses or different stress combinations). This involves identifying common genes and response pathways using existing research results or novel methodologies [9], thus uncovering avenues to activate stress combination signal transduction pathways [11], and implementing them in plants [23]. However, it is crucial not to overlook the examination of specific responses to stress combinations, particularly under multiple stresses. Delving into specific responses helps frame stress combinations as distinct states and offers a more comprehensive understanding of how plants react to these complex stress scenarios.

#### 5.2.2. Identification of Stress Combination Targets

Identifying and characterizing targets within plants experiencing stress combinations, refining target recognition techniques, and integrating them into breeding strategies are the primary objectives for bolstering resilience against stress combinations. Although nascent, research on plant targets under stress combinations has shown some advancements. Certain TFs that are pivotal in stress combinations could potentially serve as targets for enhancing plant resistance to these complex stress environments [23]. For instance, TFs such as MYB and those with family-specific functions are amplified under high-light and heat stress combinations, offering promising targets for enhancing plant resilience to this specific stress blend [53]. Moreover, photosystem II (PSⅡ) plays a crucial role in the combination of high-light and heat stress, possibly constituting a prospective cellular target [53]. Recent studies have underscored the intricate changes in plant metabolomes under stress combinations, positing stress-resistant metabolites as potential targets for plant breeding initiatives [40,95]. Additionally, maintaining potassium homeostasis is a critical element in plant response to stress combinations, potentially presenting a molecular pathway as a biotechnological target [96]. Stomata, a more common site of physiological change in plants, play an important role in the crosstalk between different stress response pathways and can be used as a target for biotechnology [32].

Target identification of plant responses to stress combinations is an effective means of enhancing plant tolerance; however, research in this area remains limited and requires more in-depth studies and further validation. Using varieties with different sensitivities to stress combinations, exploring possible mechanisms, and breeding varieties with enhanced tolerance to stress combinations may be an effective strategy [22,97].

#### 5.2.3. Adaptive Genetic Response

In nature, plants experience various stress combinations that can occur simultaneously or successively. Current research has predominantly focused on simultaneous stress. However, climate change is elevating the frequency of sequential occurrences. Although most simultaneous stress combinations adversely affect plant growth and development, sequential events generally do not affect plants the same way [8]. These successive stresses may lead to phenotypic plasticity [29], epigenetic changes [98], stress memory, and a series of adaptive responses that improve plant tolerance to stressful environments. The occurrence of successive stresses allows plants to not only mitigate repeated stress damage but also to better respond to other stresses [9]. The occurrence of one stress event may cause short- or long-term changes in the antioxidant or transcriptional system of plants, triggering stress memory and increasing stress tolerance.

In addition to the inherent adaptive responses of plants to stress combinations, systemically acquired acclimatization may serve as an effective method for alleviating plant damage from such stress scenarios. Research indicates that plants possess the ability to amalgamate various stress signals from distinct leaves, thereby inducing systemically acquired acclimatization in response to stress combinations [45,46]. This empowers plants to swiftly adapt to forthcoming stress combinations, promoting better survival rates [43]. This strategy has been successfully implemented in crops, such as *Oryza sativa* and *Triticum aestivum*, resulting in concurrent improvements in yield and resilience [38]. These findings suggest that inducing acclimatization in plant systems by mimicking environmental stress stimuli is a promising strategy for mitigating the detrimental effects of environmental stress on plants.

#### 5.2.4. Use of High Technology

In our rapidly evolving world, an array of advanced technologies is emerging that offer effective avenues to alleviate plant damage caused by stress combinations. Among these approaches, machine learning is one of the most efficient and promising methods [99]. Advances in histological technologies have generated a large amount of data on plant responses to stress combinations, and machine learning methods can be used to predict functional genes [100] and gene regulation from these data [101]. Integrated application and deeper mining of these data in combination with meta-analysis, CRISPR/Cas9 gene editing, and nanotechnology can improve our understanding of stress combinations [27]. Precision agriculture is the future direction of agricultural development, and the use of remote sensing data and machine learning, coupled with improved phenotyping and breeding methods, allows for the rapid discrimination of resistance phenotypes in plants through high-throughput methods [102], predicting plant pest and disease risks [103,104,105], controlling weeds [106,107], identifying environmental and nutrient status [108], and monitoring plant growth [109]. The combined use of these can accelerate the development of resistant plant varieties, favoring plant growth efficiency and tolerance to stress combinations [63].

Harnessing cutting-edge tools such as machine learning, multi-omics research, gene editing, nanotechnology, remote sensing monitoring, and imaging, along with the integration of novel concepts and research methodologies from diverse disciplines, is crucial. This approach enables a comprehensive understanding of plant responses to stress combinations, thereby facilitating the development of plant varieties with heightened tolerance, improved crop yields, and effective climate change mitigation strategies.

## 6. Closing Remarks

Plants provide essential guarantees for human survival and development; however, owing to the complex climate change in the future, plants may experience more complex and unpredictable growth environments, posing novel challenges to ecological diversity and food security. For example, a single incidental increase in stressors under a stress combination can lead to a sudden drop in plant survival, and researchers are increasingly focusing on studying plant responses to stress combinations. Therefore, understanding the current research focus and methodologies being used to explore future research directions regarding enhanced stress combinations is crucial. Researchers have actively used various technologies and research methods to study the responses of plants to stress combinations and some important mechanisms; however, many problems remain. We need to find key entry points and efficient research methods and make efforts from various aspects to make plants cope better with climate change.

## Data Availability

Data sharing not applicable.
